# Trauma-informed care for women who are forced migrants: a qualitative study among service providers

**DOI:** 10.1177/14034948241237591

**Published:** 2024-03-14

**Authors:** Linda Jolof, Patricia Rocca, Tommy Carlsson

**Affiliations:** 1 The Swedish Red Cross Treatment Centre for Persons Affected by War and Torture, Sweden; 2 Department of Health Sciences, The Swedish Red Cross University, Sweden; 3 Department of Women’s and Children’s Health, Uppsala University, Sweden

**Keywords:** Armed conflicts, internal displacement, refugees, trauma-informed care, women

## Abstract

**Aims:**

The aim was to explore service providers’ perspectives on trauma-informed care for women who are forced migrants.

**Methods:**

Service providers (*n*=32) employed at one of six centres providing trauma-informed care for forced migrants were recruited by way of managers. Audio-recorded and transcribed semi-structured focus group discussions were analysed with systematic text condensation.

**Results:**

The analysis revealed exposure to gender-based violence and abuse within patriarchal structures as the main challenges for women. Participants recognised remarkable strength and resilience among women. A range of structural, psychosocial and individual barriers to trauma-informed care were addressed. While trauma-informed care was considered to have the potential to improve the health for many women, participants articulated room for improvement in the competence of service providers and the conditions impacting women’s opportunities to access support.

**Conclusions:**

**Violence, abuse and oppression against forced migrant women severely impact their health and possibilities of accessing support. Services providing trauma-informed care for forced migrants need to empower women, and carefully consider gender-related aspects impacting women’s opportunities to access and utilise trauma-informed care. To ensure that women who need support access it, trauma-informed services should work with outreach efforts, ensure competence development among providers, counteract practical barriers and coordinate with health and social services.**

## Background

Forced migration, an involuntary or coerced movement as a result of persecution, conflict, violence or human rights violations, is a pressing global concern severely impacting the health and wellbeing of millions each year [1]. Many displaced persons are women, who encounter unique challenges [2] and are exposed to gender-based risks [2–4]. These women are at a high risk of experiencing psychological distress [5, 6] and somatic illness [6, 7]. Post-traumatic stress disorder [8] and low quality of life [9] is prevalent among these women, who encounter inequalities and unmet needs of support from health services [7, 10]. Social disadvantages impacting the health and wellbeing of migrant women call attention to the importance of adequate support [11, 12]. Many countries have implemented specialised units providing multidisciplinary trauma-informed care for those with traumatic experiences [13]. In-depth knowledge of the challenges encountered by health professionals supporting these women is necessary, to gain insights into how trauma-informed care can be further tailored and improved [7, 10]. Studies can generate further context-based knowledge, informing health professionals and decision makers about clinical considerations.

## Aim

The aim of this study was to explore service providers’ perspectives on trauma-informed care for women who are forced migrants.

## Methods

### Study design and context

This qualitative study explored perspectives among service providers working at treatment centres in Sweden. A qualitative and inductive approach was utilised to remain open-ended towards information-rich data [14] collected through focus group discussions [15]. The study was approved by the Swedish Ethical Review Authority (approval number 2022-03636-01). All participants provided oral and written informed consent. None of the authors worked as managers at any centre, and informed consent was collected without manager involvement.

In Sweden, forced migrants have the right to healthcare that cannot be deferred. Most clients at the centres are admitted by way of referrals from health services, but some also accept self-referrals. Clients are offered a multi-disciplinary approach involving individual and/or group-based sessions with professions such as psychologists, physiotherapists and social workers. All are offered and can decline interpreter services.

### Participant recruitment and sample characteristics

Service providers employed at one of six centres were recruited through clinical managers, who distributed study information to professionals, and those expressing an interest were contacted by the research team. In total, 32 service providers were included (centre A: eight participants; centre B: nine participants; centre C: three participants; centre D: four participants; centre E: four participants; centre F: four participants). Participants represented psychologists (*n*=18), psychotherapists (*n*=4), social workers (*n*=4), nurse assistants/administrators (*n*=2), physiotherapists (*n*=2), nurses (*n*=1) and service improvement officers (*n*=1). The median time working in each current service was 1.5 years (range 0–9 years). In total, 28 participants were women and four were men. The median age was 40 years (range 27–65 years). There was a range of ethnicities represented.

### Data collection

Seven semi-structured focus group discussions were conducted (five face-to-face discussions with 25 participants and two by way of a digital video conferencing tool with seven participants), utilising a topic guide developed a priori addressing: women’s specific support needs; how women experience treatment; what is needed to provide adequate support; and suggestions for improvements. Follow-up and clarifying questions were asked when needed. The length of the discussions ranged between 54 and 68 (median 63) minutes. All discussions were audio recorded and transcribed verbatim.

### Data analysis

The discussions were analysed with systematic text condensation, a cross-case thematic qualitative analysis [16]. The analysis was iterative and inductive, involving the following steps: (a) identified preliminary themes and engaged in joint discussions until agreement was reached; (b) material was coded into code groups and subgroups; (c) condensates acting as artificial quotes were produced and illustrative quotes were identified; (d) synthesised statements summarising the content were written; and (e) category headings were produced. All authors engaged in the identification of preliminary themes and category headings. The first two authors were responsible for producing initial drafts of steps (b) to (d), which were scrutinised by the last author and discussed in repeated joint discussions. The first two authors are service providers (psychologist and physiotherapist) working at one of the trauma centres, while the last author is an associate professor, researcher and specialist nurse-midwife with no affiliation to the trauma centres.

## Results

The analysis revealed exposure to gender-based violence and abuse within patriarchal structures as a main challenge for women forced migrants (hereafter referred to as ‘women’). Participants recognised that women show remarkable strength and resilience. In the discussions, participants highlighted a range of structural, psychosocial and individual barriers hindering access to services providing adequate trauma-informed care. While participants acknowledged that trauma-informed care has the potential to improve the health and wellbeing for women, there is room for development of competence and structural conditions that would improve their opportunities to utilise the support.

### Women are exposed to gender-based violence and abuse within patriarchal structures that impact their health while simultaneously showing strength and resilience

Participants acknowledged that women carry various experiences of violence, spanning from general to gender-based violence throughout their migration trajectories. The risk of being exposed to sexual violence was highlighted. Participants described that sexual violence affects women throughout various contexts. The exposure to violence and abuse against women was considered often to persist in the resettlement phase – for example, when forced to engage in sexual acts. Violence against women could also manifest in domestic settings, with potential long-term effects leading to considerable health-related consequences.

Women need to use sex as a form of payment in different ways than men do. They need to use it partly to obtain assistance in fleeing, but also along the way of the migration. In certain cases, when they arrive here and need to have somewhere to stay, they also pay with sex. (Participant 1, administrator at centre A)

Women’s exposure to intersecting disadvantages related to gender and migration was highlighted, including that women often have a low position within their families and experience structural discrimination. Participants described that women carry experiences of societal oppression stemming from patriarchal structures in their countries of origin and in the host country, which was considered to contribute to challenges for women in making their voices heard and seeking support. Women were also considered at risk of not prioritising their own needs of support, when worrying about the needs of family members.

Almost all women have lived under patriarchal oppression, which means that their problems are intertwined with what has happened in terms of torture, displacement, war, as well as experiences from their childhood. The challenging circumstances that they bring with them make it difficult for them to express their own needs. (Participant 2, psychologist at centre A)

According to the participants, women undergoing trauma-informed care display a range of health-related burdens. Various manifestations and symptoms of psychological distress were described, including anxiety, concentration problems, dissociation, grief, guilt, limited patience, post-traumatic stress, sadness, shame, sleep difficulties and trust issues. Fears were considered to impact the lives and wellbeing of some women, including a fear of others finding out about their past experiences and a fear of men. Participants also described that women often experience physical, at times chronic, pain conditions. Loneliness and social isolation were also emphasised.

Establishing trust can be challenging. If they have trust issues, it can take some time for them to dare to trust others fully. Perhaps they feel secure in their relation to immediate family, but quite insecure in and experience difficulties forming relationships outside of the immediate family. It can be even more challenging for them if there is a lack of trust and because of insecurities related to PTSD [post-traumatic stress disorder]. (Participant 3, psychologist at centre B)

Participants highlighted the considerable strength displayed by women, who were considered to show remarkable resilience by resisting oppression and standing up for their rights. Participants described how women keep their families together under difficult circumstances, and constantly have their children’s best interests at heart. Women were seen to value relationships, be loyal and possess a strong ability to build community. One participant described that women become frustrated when being seen as victims despite demonstrating courage and resilience.

A very traumatised mother was able to accommodate eight children and move from apartment to apartment with relatives while living in homelessness. She managed to accommodate eight children! (Participant 4, psychotherapist at centre B)

### Structural, psychosocial and individual barriers make it difficult for women to seek and access adequate health services and support

Participants highlighted various structural barriers to health services providing adequate trauma-informed care, which were considered too few in relation to the number of women in need of support. According to participants, women encounter challenges when trying to navigate the Swedish healthcare system. Further, participants mentioned that health services oftentimes are not able successfully to identify women exposed to violence, even when women gain contact with health services. The lack of identification of women in need of trauma-informed care was attributed to low staffing in health services and a general underutilisation of interpreter services. Potential sources for a lack of access included incorrect assessments and a lack of self-referral options. Barriers within the organisational structure of services providing trauma-informed care were discussed, including constraints in criteria for admission and long waiting lines. Participants expressed that the healthcare system and society lack a general knowledge about trauma-informed care.

I think there are too high thresholds before women can access the healthcare system and make their voices heard. I assist many patients who come here with current healthcare contacts. They have tried accessing support by going to a primary healthcare centre but haven’t understood how to make a phone call. (Participant 4, nurse at centre B)

Psychosocial circumstances hindering access to trauma-informed care were described. A lack of education among women and limited participation in public life were considered potential barriers. Challenges with transportation to health services, financial constraints, a lack of educational opportunities, difficulties finding employment and poor health among family members were recognised as additional barriers. Participants highlighted limited access to childcare and a fear of having their children taken from them as gender-based barriers further hindering access to treatment. Oppression and violence enacted by persons in women’s proximity were recognised as additional barriers, as well as undergoing a stressful asylum process and having family members or relatives in areas affected by armed conflicts.

Not wanting that their partner will find out that they are undergoing treatment, or worrying about that possibility, can be a barrier for seeking healthcare. Another significant challenge can be if women have children and are expected to take care of them, which becomes a hindrance. (Participant 5, psychologist at centre C)

Internal processes within women were seen as further barriers to trauma-informed care. Trust issues towards health services and concerns about confidentiality were addressed, alongside shame and not believing they deserve treatment. Participants recognised various fears that can act as barriers, including fears of encountering male service providers and interpreters, needing to interact with interpreters who are compatriots, and needing to travel to the service after dark. Women with somatic conditions, pain conditions and multiple simultaneous healthcare contacts were recognised as at risk of not being able to focus during trauma-informed care. Resistance to change, insufficient communication skills, lack of motivation and exhaustion were also acknowledged as potential sources for the inability to undergo treatment.

I have met many women who have been exposed to abuse, which can be very shameful to start talking about. For several of those I have met, no one knew about them undergoing the treatment. Especially not their husbands, out of a fear of being abandoned or rejected if [their husbands] would find out. (Participant 6, psychologist at centre C)

### There are opportunities to provide women with adequate trauma-informed care, but more specific knowledge and collaboration is needed

The significance of adequate communication and establishing positive relationships between service providers and women were emphasised. Participants called attention to many women expressing gratitude and attachment towards providers. Further, they emphasised the importance of applying a flexible, individualised and person-centred approach. Trauma-informed care was acknowledged to have the potential to improve the health and wellbeing of women, including reducing symptoms of post-traumatic stress and improving quality of life.

I think the most important thing is that we listen to them, really. They come here because they want to talk, because they can’t talk when they are home. They can’t talk to their friends. I think that listening to them is the most important thing. (Participant 7, psychologist at centre D)

Participants highlighted the importance of structural components when providing trauma-informed care for women. Ensuring that service providers are provided with opportunities to develop their competence in related areas was underscored – for example, domestic violence, developmental trauma, patriarchal oppression, cultures, norms, sexual health and reproductive health. The value of teamwork and coordination was highlighted, including the provision of legal assistance and collaborations with other health services, organisations, municipalities and authorities. When discussing ways to improve the care further, participants suggested making sure that information is available in multiple languages, offering digital remote treatment for those who need it, offering group-based activities to a greater extent and developing treatment in collaboration with women. Participants also called attention to a need for improved awareness about available trauma-informed services among professionals working within authorities and general health services.

I can experience significant knowledge gaps, including about the type of care they would prefer in a context like this and what experiences exist within that patient group. What has been effective for them and what has been helpful. (Participant 8, psychologist at centre A)

Complex psychosocial situations were recognised to have an impact on the outcomes of treatment. To help women benefit from treatment, participants called attention to the importance of having a social worker and/or a case manager supporting these women and handling the coordination between various health services. While participants expressed that women appreciate the psychosocial support and family activities offered within their services, they also emphasised the need for further supporting women’s parenting roles. The importance of addressing the support needs of the family was recognised, including offering family-oriented treatment activities. A suggestion was to provide childcare services during treatment to improve women’s access to support and to reduce potential treatment dropouts.

There’s a lot about the practical aspects, such as being able to offer travel reimbursement and childcare. Either by providing childcare here or having volunteers who can come regularly to help with childcare, or by talking to preschools about giving this person an extra 2 hours so she can come here for treatment. (Participant 9, service improvement officer at centre E)

## Discussion

This study explored service providers’ perspectives on trauma-informed care for women who are forced migrants. Even though host countries have a responsibility to ensure adequate support for these women, participants acknowledged several barriers to trauma-informed care. Insufficient awareness of available support and fear of stigma are significant contributors to poor utilisation of mental health services among refugees and asylum seekers [17]. Targeted outreach to reach forced migrants has been highlighted as a strategy to increase their utilisation of available health services [18]. Consequently, services providing support for these women should continue working towards increased awareness of available support [19]. Previous studies highlight the importance of effective coordination between health services and sufficient competence on a broad level to achieve successful referrals [20]. In line with previous research, our participants emphasised gender hierarchies and responsibilities in daily life as further barriers [21]. Services providing trauma-informed care should consider how they can enhance women’s opportunities to access support by implementing actions that will counteract these practical barriers. We encourage efforts that address coordination between different health services, collaboration with childcare services, and providing women free transportation to treatment appointments.

Participants highlighted structural disadvantages, oppression, violence and abuse against women, severely impacting their health and wellbeing. Violence against women and girls, including discrimination, is a global public health crisis requiring strong actions [22]. Throughout their migration trajectories, refugee women are exposed to violence and abuse [2, 23]. Too many refugee women are daily exposed to sexual violence enacted by a range of various perpetrators, leading to impactful health-related and social consequences [24]. Our participants articulated a need for competence development in related topics, including domestic violence and patriarchal oppression. Indeed, previous studies have found that health professionals need more training in the management of clients exposed to domestic violence [25, 26]. Our findings highlight the importance of empowering women who are exposed to violence, abuse and oppression. Further, the findings suggest a need to develop interventions promoting competence development among professionals providing trauma-informed care for women who are forced migrants.

There are methodological limitations that need to be considered when interpreting the findings. The participants were recruited from six centres providing trauma-informed care for forced migrants. The centres are geographically dispersed throughout Sweden, and the participants represent a range of professions. Two authors, employed at one of the centres (psychologist and physiotherapist), collected data and contributed to the analysis. While we cannot dismiss the possibility that the data collection and analysis involved some bias, we strived for neutrality by having the last author plan the data collection, collect a proportion of the data and scrutinise the stepwise process in the analysis from an outsider’s perspective. Preconceptions and prejudices among the participants probably influenced the findings, which needs to be taken into consideration when interpreting the results.

## Conclusions

Violence, abuse and oppression against forced migrant women severely impact their health and the possibilities of accessing support. Services providing trauma-informed care for forced migrants need to empower women, and carefully consider the gender-related aspects that impact women’s opportunities to access and participate in adequate support. Trauma-informed care needs to take into consideration the violence, abuse and structural oppression against women. To ensure that women who need trauma-informed support access and utilise it, services and research should consider outreach efforts, competence development, practical barriers and effective coordination with health and social services.
